# CRISPR-Cas12a-assisted nucleic acid detection

**DOI:** 10.1038/s41421-018-0028-z

**Published:** 2018-04-24

**Authors:** Shi-Yuan Li, Qiu-Xiang Cheng, Jing-Man Wang, Xiao-Yan Li, Zi-Long Zhang, Song Gao, Rui-Bing Cao, Guo-Ping Zhao, Jin Wang

**Affiliations:** 1 Key Laboratory of Synthetic Biology, Institute of Plant Physiology and Ecology, Shanghai Institutes for Biological Sciences, Chinese Academy of Sciences, 200032 Shanghai, China; 2 Shanghai Tolo Biotechnology Company Limited, 200233 Shanghai, China; 3 State Engineering Laboratory of Medical Key Technologies Application of Synthetic Biology, Shenzhen Second People’s Hospital, The First Affiliated Hospital of Shenzhen University, Shenzhen, China; 4 Shanghai International Travel Healthcare Center, Shanghai Entry-Exit Inspection and Quarantine Bureau, 200335 Shanghai, China; 5 Jiangsu Co-innovation Center for Prevention and Control of Important Animal Infectious Diseases and Zoonoses, Key Laboratory of Avian Bioproducts Development, Ministry of Agriculture, College of Veterinary Medicine, Yangzhou University, Yangzhou, Jiangsu 225009 China; 6 Key Laboratory of Animal Diseases Diagnostic and Immunology, Ministry of Agriculture, College of Veterinary Medicine, Nanjing Agricultural University, Nanjing, Jiangsu 210095 China; 7 Department of Microbiology and Li Ka Shing Institute of Health Sciences, The Chinese University of Hong Kong, Prince of Wales Hospital, Shatin, New Territories, Hong Kong SAR, China

Dear Editor,

Today, the need for time-effective and cost-effective nucleic acid detection methods is still growing in fields such as human genotyping and pathogen detection. Using synthetic biomolecular components, many methods have been developed for fast nucleic acid detection^1–3^; however, they may not be able to satisfy specificity, sensitivity, speed, cost and simplicity at the same time. Recently, a very promising CRISPR-based diagnostic (CRISPR-Dx) (namely SHERLOCK) was established, which was based on the collateral effect of an RNA-guided and RNA-targeting CRISPR effector, Cas13a^4^. SHERLOCK is of high sensitivity and specificity, and is very convenient in detection of target RNA. However, to detect DNA sequences, in vitro transcription of DNA to RNA must be conducted prior to the SHERLOCK test, which could be inconvenient.

In a recent study, we found that Cas12a, which belongs to the class 2 type V-A CRISPR-Cas system^5^, performed collateral cleavage on non-targeted ssDNAs upon the formation of the Cas12a/crRNA/target DNA ternary complex^6^. Here, with the employment of this feature, we used a quenched fluorescent ssDNA reporter (e.g., HEX-N12-BHQ1 in Supplementary Table S1) as the probe, and developed HOLMES (an one-HOur Low-cost Multipurpose highly Efficient System), which could be used for fast detection of target DNA as well as target RNA. In HOLMES, if a target DNA exists in the reaction system, the Cas12a/crRNA binary complex forms a ternary complex with the target DNA, which will then *trans*-cleave non-targeted ssDNA reporter in the system, illuminating the HEX fluorescence (or any other fluorescence) (Fig. 1a).

**Fig. 1:**
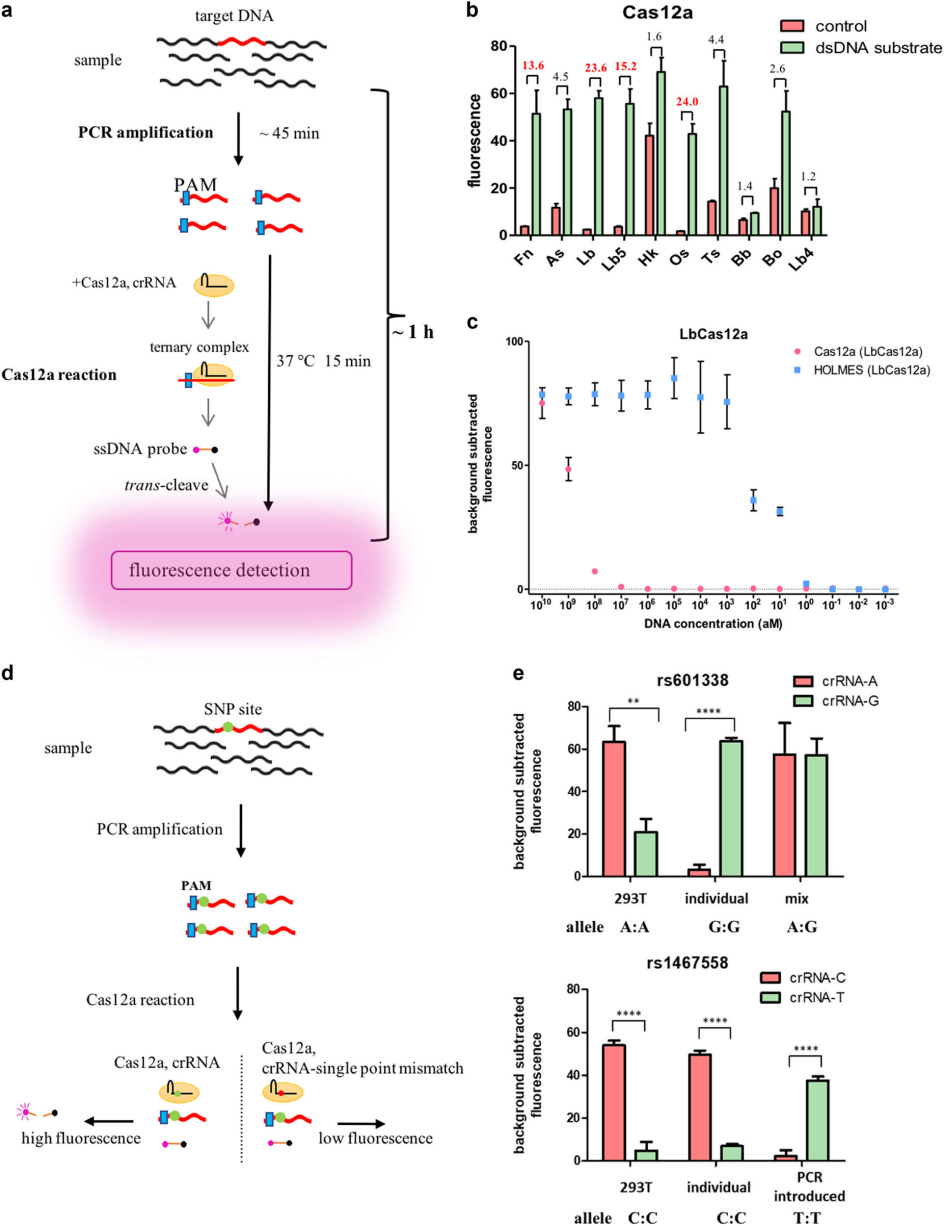
HOLMES is a rapid, simple and efficient method for nucleic acid detection. **a** An illustration of HOLMES. To detect a target DNA, specific amplification of the target DNA by either PCR or other isothermal amplification methods will be performed, and a crRNA guide sequence is specially designed, targeting a region in the target DNA. The PAM sequence can be designed on the primers and introduced during amplification. After that, the amplicon was mixed with the Cas12a/crRNA complex, and a ternary complex forms if the target DNA exists. Upon the formation of the ternary complex, the quenched fluorescent ssDNA reporter is *trans*-cleaved, illuminating the fluorescence. **b** Comparison of the signal-to-noise values of *trans*-cleavage by ten Cas12a proteins from different species. The reaction system included Cas12a, crRNA (crRNA-T1), target DNA (pUC18-T1) and quenched fluorescent ssDNA (HEX-N12-BHQ1), and the target DNA was omitted in the negative control. The signal-to-noise values were labeled and values larger than 10 were shown in red (*n* = 3 technical replicates; bars represent the mean ± SEM). Fn *Francisella tularensis*; As *Acidaminococcus sp*. BV3L6; Lb *Lachnospiraceae bacterium* ND2006; Lb5 *Lachnospiraceae bacterium* NC2008; HK *Helcococcus kunzii* ATCC 51366; Os *Oribacterium sp*. NK2B42; Ts *Thiomicrospira sp*. XS5; Bb *Bacteroidales bacterium* KA00251; Bo *Bacteroidetes oral taxon* 274 str. F0058; Lb4 *Lachnospiraceae bacterium* MC2017. **c** Detection sensitivity of Cas12a alone or Cas12a combined with PCR amplification (i.e., HOLMES). Serially diluted pUC18-T1 plasmid was employed as the target dsDNA. (*n* = 3 technical replicates; bars represent the mean ± SEM). **d** Schematic of human SNP genotyping by HOLMES. The amplification of a target DNA containing the SNP locus is almost the same as described in Fig. 1a, and design of primers and introduction of the PAM site are detailed in Supplementary Figure S3. To detect an SNP, more than one crRNA is needed, targeting different genotypes. **e** HOLMES correctly genotyped different human SNP loci in HEK293T, a candidate individual, and the PCR-generated templates (*n* = 3 technical replicates; two-tailed Student’s *t*-test; **p < 0.01; ****p < 0.0001; bars represent the mean ± SEM). Genotypes verified by Sanger sequencing were annotated below each plot, and the results of other SNP loci could be found in Supplementary Figure S4a

We ever purified ten Cas12a proteins (Supplementary Table S3) and found all showed the ssDNA *trans*-cleavage activity^6^. To find the most suitable Cas12a for HOLMES (i.e., with high signal-to-noise ratios), we tested all ten Cas12a proteins and found *Lachnospiraceae bacterium* ND2006 Cas12a (LbCas12a), *Oribacterium sp*. NK2B42 Cas12a (OsCas12a), *Lachnospiraceae bacterium* NC2008 Cas12a (Lb5Cas12a) and *Francisella tularensis* Cas12a (FnCas12a) showed good performance, among which LbCas12a was chosen for the following studies (Fig. 1b). To determine the sensitivity of HOLMES, we titrated target DNA, and found the minimum detectable concentration for Cas12a-crRNA was approximately 0.1 nM; however, when combined with PCR, the detectable concentration could be as low as 10 aM (Fig. 1c), which was comparable to the SHERLOCK system^4^ and was better than PCR alone or quantitative PCR using the SYBR Green method (Supplementary Figure S1). Therefore, to achieve higher sensitivity, PCR amplification was employed in the HOLMES test thereafter.

To test whether HOLMES could discriminate single-base differences, we made point mutations at different positions in the target DNA sequence, including both the PAM region and the guide sequences (Supplementary Figure S2a). When a full length of crRNA guide sequence (24-nt crRNA, Supplementary Table S2) was used, we found mutations in either the PAM sequences or the region of the 1st–7th bases of the guide sequence resulted in clear decline of the fluorescence signal; however, no significant difference was observed when the mutation was within the region of the 8th–18th bases (Supplementary Figure S2b), which was highly consistent with the previous report that the 5′-end seed region in the crRNA guide sequence was extremely important for Cas12a recognition^7^. In addition, based on our previous findings^8^, Cas12a with a reduced length of crRNA guide sequence showed higher cleavage specificity. Therefore, we then tested shorter guide sequences, and found point mutations within a larger region (1st–16th bases) resulted in more than 2-fold difference in fluorescence signals for both 16-nt and 17-nt crRNA guide sequences (Supplementary Figure S2b), suggesting that shorter guide sequences might be used in HOLMES. Furthermore, considering the fact that there might exist no suitable PAM sequence nearby the SNP site, primers for PCR amplification were specially designed to introduce the PAM sequence (Supplementary Figure S3), which therefore allowed for sequence-independent detection of any single nucleotide polymorphism (SNP) sites.

We then chose a dozen of SNP loci that are related to human health and personal characteristics (Supplementary Table S4). We either extracted genomic DNA from cultured human 293T cells or collected saliva from human individuals, and then PCR amplified the target regions, followed by the HOLMES assay to distinguish alleles (Fig. 1d). The results clearly showed that HOLMES had sufficiently high specificity to determine both homozygous and heterozygous genotypes (Fig. 1e and Supplementary Figure S4a). We also collected nineteen volunteers’ saliva samples to detect the SNP rs1014290, which is related to gout risk, and proved that HOLMES could be used to rapidly and easily detect human SNP genotypes (Supplementary Figure S4b).

Moreover, HOLMES could also be used to detect DNA viruses (e.g., pseudorabies virus (PRV), Supplementary Figure S5a) and RNA viruses (e.g., Japanese encephalitis virus (JEV), Supplementary Figure S6a), and the sensitivity for both could be as low as 1–10 aM (Supplementary Figures S5b and S6b). For JEV, total RNA was first extracted and then reverse transcribed into cDNA before being detected by HOLMES. Because of the high sensitivity, HOLMES successfully detected PRV virus in both the PRV-infected cells and the culture supernatant (Supplementary Figure S5c). In addition, the high specificity of HOLMES also enabled it to distinguish between virus strains. For example, the PRV Ra classical strain, the cmz variant strain and the Bartha-K61 vaccine strain were easily discriminated by the gE46 site (Supplementary Figure S5d and S5e). Similarly, the JEV NJ2008 strain and the live-attenuated vaccine strain SA14-14-2 were well differentiated by the site E138 (Supplementary Figure S6c and S6d).

The “SHERLOCK” nucleic acid detection system was recently established with the employment of the “RNA collateral effect” of Cas13a and an isothermal amplification method^6^. Although both HOLMES and SHERLOCK show attomolar detection sensitivity and can be used to detect both DNA and RNA targets, this study indicates that HOLMES may have advantages in DNA detection, while SHERLOCK is more convenient for RNA detection. In addition, isothermal amplification methods (e.g., the recombinase polymerase amplification (RPA) and loop-mediated isothermal amplification (LAMP)) can also be used although rapid PCR amplification was used in HOLMES in this study. Similar to SHERLOCK, HOLMES requires no expensive reagents and no special instruments, making it low cost and easily accessible for nucleic acid detection. In addition to the medical applications described above, HOLMES may also be used for a variety of applications that require rapid detection of nucleic acids, including monitoring foods and the environment.

(While this manuscript has been ready to submit to *Cell Discovery*, two pieces of work were published on *Science*, both of which described the use of the Cas12a *trans*-cleavage activity on ssDNAs for nucleic acid detection^9, 10^.)

## Electronic supplementary material

Supplementary Information

## Electronic supplementary material

**Supplementary Information** accompanies the paper at (10.1038/s41421-018-0028-z).
