# Author Correction: CRISPR-Cas12a-assisted nucleic acid detection

**DOI:** 10.1038/s41421-019-0083-0

**Published:** 2019-03-12

**Authors:** Shi-Yuan Li, Qiu-Xiang Cheng, Jing-Man Wang, Xiao-Yan Li, Zi-Long Zhang, Song Gao, Rui-Bing Cao, Guo-Ping Zhao, Jin Wang

**Affiliations:** 1 Key Laboratory of Synthetic Biology, Institute of Plant Physiology and Ecology, Shanghai Institutes for Biological Sciences, Chinese Academy of Sciences, 200032 Shanghai, China; 2 Shanghai Tolo Biotechnology Company Limited, 200233 Shanghai, China; 3 State Engineering Laboratory of Medical Key Technologies Application of Synthetic Biology, Shenzhen Second People’s Hospital, The First Affiliated Hospital of Shenzhen University, Shenzhen, China; 4 Shanghai International Travel Healthcare Center, Shanghai Entry-Exit Inspection and Quarantine Bureau, 200335 Shanghai, China; 5 Jiangsu Co-innovation Center for Prevention and Control of Important Animal Infectious Diseases and Zoonoses, Key Laboratory of Avian Bioproducts Development, Ministry of Agriculture, College of Veterinary Medicine, Yangzhou University, 225009 Yangzhou, Jiangsu China; 6 Key Laboratory of Animal Diseases Diagnostic and Immunology, Ministry of Agriculture, College of Veterinary Medicine, Nanjing Agricultural University, 210095 Nanjing, Jiangsu China; 7 Department of Microbiology and Li Ka Shing Institute of Health Sciences, The Chinese University of Hong Kong, Prince of Wales Hospital, Shatin, New Territories, Hong Kong SAR, China

**Correction to:** **Cell Discovery** (2018)4:20

10.1038/s41421-018-0028-z

published online 24 April 2018

In the original version of the Supplementary Information file associated with this article, the site ‘rs5082’ was incorrectly given and should be corrected to ‘rs5028’.

In detail, oligo names of ‘rs5082-F, rs5082-F-T, rs5082-R, T7-crRNA-rs5082-T, T7-crRNA-rs5082-G’ in Supplementary Tables S1 and S2 should be corrected to ‘rs5028-F, rs5028-F-T, rs5028-R, T7-crRNA-rs5028-T, T7-crRNA-rs5028-G’, and the SNP site of ‘rs5082’ in Supplementary Figure S4a should be corrected to ‘rs5028’. In Supplementary Table S4, the SNP site of ‘rs5082’ should be corrected to ‘rs5028’, the gene of ‘APOA2’ should be corrected to ‘SLC4A1’ and the category of ‘Saturated fat consumption and weight gain’ should be deleted.

