# Supplementary material for: CRISPR-Cas12a-assisted nucleic acid detection
Source: Cell Discov. 2018 Apr 24;4:20. doi: 10.1038/s41421-018-0028-z (PMC5913299; doi:10.1038/s41421-018-0028-z)
Supplement: Supplementary file 1 — Supplementary Information [file 41421_2018_28_MOESM1_ESM.pdf]

Supplementary information

Materials and Methods

Generation of dsDNA targets

Template pUC18-T1 and primers (Target-T1-F and 1013-cexu-R) were used for the detection of HOLMES sensitivity in both Figure 1c and Supplementary Figure 1. To construct plasmid pUC18-T1, PCR amplification was performed with primers pUC18-T1-F and pUC18-T1-R (Supplementary Table S1), employing plasmid pUC18 as the template, and the PCR product was then self-ligated in the presence of T4 DNA ligase (Tolo Biotech.), T4 PNK (Tolo Biotech.) and DpnI (NEB). In Supplementary Figure S2, target fragments were amplified with 1260(R) and its single-point mismatch primer (Supplementary Table S1) with pUC18-T1 as the template, followed by the purification and dilution of the fragments to the same concentration (50 ng/μL).

Transcription of crRNAs

The transcription templates were prepared through annealing of the synthesized oligonucleotides with T7-crRNA-F (Supplementary Table S2), following the same procedures as previously described[1,2]. Then, crRNAs were synthesized using a T7 High Yield Transcription Kit (Thermo Fisher Scientific), and the reaction was performed at 37 °C overnight (approximately 16 h). RNA was purified using the RNA Clean & Concentrator™-5 (Zymo Research) and quantified with the NanoDrop 2000C (Thermo Fisher Scientific).

Extraction of genomic DNA, viral DNA and viral RNA

HEK293T cells were grown at 37 °C under 5% CO_2 atmosphere in Dulbecco's modified Eagle's

medium (Invitrogen, Carlsbad, CA, USA) supplemented with 10% heat-inactivated foetal calf serum (FCS). Genomic DNA of HEK293T cells was extracted by using TIANamp Genomic DNA Kit (TIANGEN Biotech), and 10 ng of DNA was used for PCR reactions. PRV DNA virus or JEV RNA virus was extracted from viral fluids using the MiniBEST viral RNA/DNA Extraction kit (TaKaRa) according to the manufacturer's protocol.

HOLMES method

(1) PCR

PCRs were performed by KOD FX (ToYoBo) with the primers listed in Supplementary Table S1, and 1 nM purified DNA or 1 μL of saliva sample was used as the template, following the program: initial denaturation at 95 °C for 2 min, then 98 °C for 10 s, 60 °C 10 s, and 68 °C 10 s for 35 cycles. For the RNA virus (JEV), the first-strand cDNA was synthesized with reverse transcription using PrimeScript™ 1st Strand cDNA Synthesis Kit (TaKaRa) with a random hexanucleotide primer before being employed for PCR amplification.

(2) Cas12a detection

LbCas12a was used for the HOLMES cleavage assays unless mentioned otherwise. Cas12a detection was performed at 37 °C in NEB buffer 3 or 3.1 for 15 min, employing 250 nM Cas12a, 500 nM synthesized crRNA, target DNA (unless stated, 10 ng purified or 1 μL PCR reaction mixture), 500 nM collateral ssDNA (quenched fluorescent DNA reporter HEX-N12-BHQ1) and 10 U RNase inhibitor (TaKaRa) in a 20-μL volume. Reactions were stopped by heating at 98 °C for 2 min. Fluorescence emission was excited at 535 nm and detected at 556 nM using a Varioskan Flash from Thermo Fisher Scientific, and reactions with no target DNA were taken as the background.

(3) Optimization of crRNA guide sequences in SNP detection

Target T1 was employed for single nucleotide mutation analysis, and was point mutated in regions including both the PAM sequence and the 1st -18th base positions of the guide sequence. Signals between the wild-type and mutated T1 sequences were compared using crRNAs of different guide lengths. Considering the overall signal intensity and the difference between the wild-type and the mutated sequences, we employed crRNAs with 15-nt to 17-nt guide sequences for SNP detection in this study. Specifically, crRNAs with 17-nt guide sequences were first tested for their ability to discriminate single-base mismatches. If they did not work, then 16-nt (or even 15-nt) guide sequences were tested.

Real-time PCR

The sensitivity of the real-time PCR method with SYBR Green was tested by the SYBR® Premix Ex Taq ™ II reaction mixture (TaKaRa), using a dilution series of pUC18 plasmid as the template. The assay was performed with the Real-Time PCR System (StepOne Plus from Thermo Fisher Scientific).

To quantitate the DNA virus or RNA viruses using the real-time method, fragments of gD111 and E117 were first amplified from DNA and RNA viruses, using primers of gD111-F/gD111-R and E117-F/E117-R, respectively. The concentration of amplification products were determined by a NanoDrop spectrophotometer, which were then diluted to different concentrations and used as the templates to prepare the standard curves by real-time PCR with SYBR Green II. The extracted genomic DNA or reverse-transcribed cDNA was then quantitated by real-time PCR, using these standard curves.

Supplementary Table S1. Oligonucleotides used for preparation of cleavage templates in this study

Oligo names	Sequences (5′-3′)
pUC18-T1-F	tttctactgaattcggtcatagctgtttcctgtgtga
pUC18-T1-R	gttgcgataacaaaactggccgtcgttttacaacgtc
1260(R)	tgtagccgtagttaggccaccacttca
Target-T1-F	agttttgttatcgcaactttctactgaattc
1013-cexu-R	ttctgtggataaccgtattaccgc
Target-T1-F-1A	agttttgAtatcgcaactttctactgaattc
Target-T1-F-2A	agttttgtAatcgcaactttctactgaattc
Target-T1-F-3T	agttttgttTtcgcaactttctactgaattc
Target-T1-F-4A	agttttgttaAcgcaactttctactgaattc
Target-T1-F-5G	agttttgttatGgcaactttctactgaattc
Target-T1-F-6C	agttttgttatcCcaactttctactgaattc
Target-T1-F-7G	agttttgttatcgGaactttctactgaattc
Target-T1-F-8T	agttttgttatcgcTactttctactgaattc
Target-T1-F-9T	agttttgttatcgcaTctttctactgaattc
Target-T1-F-10G	agttttgttatcgcaaGtttctactgaattc
Target-T1-AAAN-F	aaaagttatcgcaactttctactgaattc
Target-T1-F-11A	agttttgttatcgcaacAttctactgaattcggtcatag
Target-T1-F-12A	agttttgttatcgcaactAtctactgaattcggtcatag
Target-T1-F-13A	agttttgttatcgcaacttActactgaattcggtcatag
Target-T1-F-14G	agttttgttatcgcaactttGtactgaattcggtcatag
Target-T1-F-15A	agttttgttatcgcaactttcAactgaattcggtcatag
Target-T1-F-16T	agttttgttatcgcaactttctTctgaattcggtcatag
Target-T1-F-17G	agttttgttatcgcaactttctaGtgaattcggtcatag
Target-T1-F-18A	agttttgttatcgcaactttctacAgaattcggtcatag
Target-T1-PAM1A-F	agtttAgttatcgcaactttctactgaattc
Target-T1-PAM2A-F	agttAtgttatcgcaactttctactgaattc
Target-T1-PAM3A-F	agtAttgttatcgcaactttctactgaattc
rs5082-F	ctgcctttgcttctacctttgcctgt
rs5082-F-T	ttgcttctacctttgcctgttctgg
rs5082-R	ttttctggctggggatggccgatgg
rs1467558-F	agcaataacactaatattgattccttcagatatggactcctttcatagta
rs1467558-F-T	ttgattccttcagatatggactcctttcatagtataacg
rs1467558-R	tgagcatcgttattcttacgcgttgtcattgaaagag
rs2952768-F	agcctgggcaacgagtgaaactctg
rs2952768-R	acaggagggacaaaggcctaagtgtcc
rs2952768-R-C	catcataggattgggaaaaggacatttcagtcattcag
rs4363657-F	agagtccttctttctcaatttttcagaataatttagtactttgggtac
rs4363657-R	cagtactgaaaaaacctgcctatcaataaaagccctagac
rs601338-F	gcttcaccggctacctttgctcct
rs601338-R	ttcacctgcaggccccgcagg
rs6869366-F	tgaaacctcacttcgtagctctgcaaactttgtact
rs6869366-F-G	ttcgtagctctgcaaactttgtactgttgac

rs6869366-R	agacttagtgatttgtttcttataaatataagtacataaattagacataggtgg
rs10034228-F	gactgtggttatgaggggaagaagtcagagatttgta
rs10034228-F-C	ttatgaggggaagaagtcagagatttgtactttgttagtgtg
rs10034228-R	gtgtggcctccatggaaacacagcg
rs9939609-F	ctaggttccttgcgactgctttgaattt
rs9939609-F-A	ttccttgcgactgctttgaatttagtgatgc
rs9939609-R	atggcttcagggtaccagctatttgcatttcag
rs838133-F	acgagaccgggttcgagctttcagg
rs838133-F-A	agaccgggttcgagctttcaggactg
rs838133-R	gtgtagaggtaccgctgccggacttg
rs17070145-F	ccagctgctccttgatctttgacct
rs17070145-R	tgcacagtgggttggcagatggaacc
rs6265-F	cgaactttctggtcctcatccaacagcttttctatca
rs6265-F-T	ttctggtcctcatccaacagcttttctatcatgtgttc
rs6265-R	aaggtggcttggcctacccaggtg
rs1014290-F	agtttccagacctcagtgcacaagatacttttctac
rs1014290-F-G	acctcagtgcacaagatacttttctacgtcatccac
rs1014290-R	agctccagtggatggaagatctttgagatccag
rs737267-F	ttcttgaacccaaactcacctggcatttaaactg
rs737267-F-T	aaactcacctggcatttaaactgtctctgtaag
rs737267-R	tgccgaggctgagttcagctactctcc
rs642803-F	ccccggctctgttggctttgagaattg
rs642803-F-C	ctctgttggctttgagaattgcctgtctgtgtc
rs642803-F-T	ctctgttggctttgagaattgtctgtctgtgtc
rs642803-R	accgatacctggcagcccttggatg
E117-F	aagcgagctgatagtagctatgtgtgcaaacaag
E117-R	atgttttctggctggattgttctcccaatcgc
E138-F	gcgattgggagaacaatccagccagaaaacatttaatac
E138-R	aaggagcattgggtgttactgtaaactttgccg
gD111-F	ggtgcgcgcacctgctgtactttatc
gD111-R	accatgagcagccccagctcgt
gE46-F	gagtccctcggccgaagtTtgggac
gE46-R	gaagttggcgccctcggacacgttca
HEX-N12-BHQ1	HEX-NNNNNNNNNNNN-BHQ1

Supplementary Table S2. Oligonucleotides used for preparation of transcription templates in this study

Oligo names	Sequences (5′-3′)
T7-crRNA-F	GAAATTAATACGACTCACTATAGGG
T7-T1-24-R	gaattcagtagaaagttgcgataaATCTACAACAGTAGAAATTCCCTATAGTGAGTCGTATTAATTTC
T7-T1-15-R	agaaagttgcgataaATCTACAACAGTAGAAATTCCCTATAGTGAGTCGTATTAATTTC

T7-T1-16-R	tagaaagttgcgataaATCTACAACAGTAGAAATTCCCTATAGTGAGTCGTATTAATTTC
T7-T1-17-R	gtagaaagttgcgataaATCTACAACAGTAGAAATTCCCTATAGTGAGTCGTATTAATTTC
T7-T1-18-R	agtagaaagttgcgataaATCTACAACAGTAGAAATTCCCTATAGTGAGTCGTATTAATTTC
T7-crRNA-rs5082-T	CCTCTTCCCAGAACAGGATCTACAACAGTAGAAATTCCCTATAGTGAGTCGTATTAATTTC
T7-crRNA-rs5082-G	CCTCTTCCCAGCACAGGATCTACAACAGTAGAAATTCCCTATAGTGAGTCGTATTAATTTC
T7-crRNA- rs1467558-T	CTGAAGCGTTATACTATATCTACAACAGTAGAAATTCCCTATAGTGAGTCGTATTAATTTC
T7-crRNA- rs1467558-C	CTGAAGCGTTGTACTATATCTACAACAGTAGAAATTCCCTATAGTGAGTCGTATTAATTTC
T7-crRNA-rs2952768-T-16nt	TTTTATCTGAATGATTATCTACAACAGTAGAAATTCCCTATAGTGAGTCGTATTAATTTC
T7-crRNA-rs2952768-C-16nt	TTTTATCTGAATGACTATCTACAACAGTAGAAATTCCCTATAGTGAGTCGTATTAATTTC
T7-crRNA-rs4363657-T	AAAAAAGAGTGAGTACCATCTACAACAGTAGAAATTCCCTATAGTGAGTCGTATTAATTTC
T7-crRNA-rs4363657-C	AAAAAAGAGTGGGTACCATCTACAACAGTAGAAATTCCCTATAGTGAGTCGTATTAATTTC
T7-crRNA-rs601338-G	GGTAGAAGGTCCAGGAGATCTACAACAGTAGAAATTCCCTATAGTGAGTCGTATTAATTTC
T7-crRNA-rs601338-A	GGTAGAAGGTCTAGGAGATCTACAACAGTAGAAATTCCCTATAGTGAGTCGTATTAATTTC
T7-crRNA-rs6869366-G	TTGGGATGTCAACAGTAATCTACAACAGTAGAAATTCCCTATAGTGAGTCGTATTAATTTC
T7-crRNA-rs6869366-T	TTGGGATGTCAAAAGTAATCTACAACAGTAGAAATTCCCTATAGTGAGTCGTATTAATTTC
T7-crRNA-rs10034228-T-16nt	CCACACTAACAAAATAATCTACAACAGTAGAAATTCCCTATAGTGAGTCGTATTAATTTC
T7-crRNA-rs10034228-C-16nt	CCACACTAACAAAGTAATCTACAACAGTAGAAATTCCCTATAGTGAGTCGTATTAATTTC
T7-crRNA-rs9939609-A	AAGTGCATCACTAAATTATCTACAACAGTAGAAATTCCCTATAGTGAGTCGTATTAATTTC
T7-crRNA-rs9939609-T	AAGTGCATCACAAAATTATCTACAACAGTAGAAATTCCCTATAGTGAGTCGTATTAATTTC
T7-crRNA-rs838133-A	CAGAAACCCACAGTCCTATCTACAACAGTAGAAATTCCCTATAGTGAGTCGTATTAATTTC
T7-crRNA-rs838133-G	CAGAAACCCACAGCCCTATCTACAACAGTAGAAATTCCCTATAGTGAGTCGTATTAATTTC
T7-crRNA-rs17070145-C	TCAGGAACAGTTGAGGTATCTACAACAGTAGAAATTCCCTATAGTGAGTCGTATTAATTTC

T7-crRNA-rs17070145-T	TCAGGAACAGTTAAGGTATCTACAACAGTAGAAATTCCCTAT AGTGAGTCGTATTAATTTC
T7-crRNA-rs6265-C	CTTTCGAACACGTGATAATCTACAACAGTAGAAATTCCCTATA GTGAGTCGTATTAATTTC
T7-crRNA-rs6265-T	CTTTCGAACACATGATAATCTACAACAGTAGAAATTCCCTATA GTGAGTCGTATTAATTTC
T7-crRNA-rs1014290-A-16nt	GTCAGTGGATGATGTAATCTACAACAGTAGAAATTCCCTATA GTGAGTCGTATTAATTTC
T7-crRNA-rs1014290-G-15nt	TCAGTGGATGACGTAATCTACAACAGTAGAAATTCCCTATAG TGAGTCGTATTAATTTC
T7-crRNA-rs737267-G-16nt	TCTTACAGAGCCAGTTATCTACAACAGTAGAAATTCCCTATA GTGAGTCGTATTAATTTC
T7-crRNA-rs737267-T	GTCTTACAGAGACAGTTATCTACAACAGTAGAAATTCCCTAT AGTGAGTCGTATTAATTTC
T7-crRNA-rs1260326-C-15nt	CTGGACTCTCACCGGATCTACAACAGTAGAAATTCCCTATAG TGAGTCGTATTAATTTC
T7-crRNA-rs1260326-T-15nt	CTGGACTCTCACCAGATCTACAACAGTAGAAATTCCCTATAG TGAGTCGTATTAATTTC
T7-crRNA-rs642803-C	CACAGACAGGCAATTCTATCTACAACAGTAGAAATTCCCTAT AGTGAGTCGTATTAATTTC
T7-crRNA-rs642803-T	CACAGACAGACAATTCTATCTACAACAGTAGAAATTCCCTAT AGTGAGTCGTATTAATTTC
T7-crRNA-E117-R	ggaagggaagcattgacacatgtgATCTACAACAGTAGAAATTCCCTATAG TGAGTCGTATTAATTTC
T7-crRNA-E138-R-A	aaatgccaacttTgtatATCTACAACAGTAGAAATTCCCTATAGTGAGT CGTATTAATTTC
T7-crRNA-E138-R-G	aaatgccaacttcgtatATCTACAACAGTAGAAATTCCCTATAGTGAGT CGTATTAATTTC
T7-crRNA-gD111-R	tggggtcgcagtcggcgtactcgaATCTACAACAGTAGAAATTCCCTATAG TGAGTCGTATTAATTTC
T7-crRNA-gE46-R	cctcggtggagaggtccATCTACAACAGTAGAAATTCCCTATAGTGAG TCGTATTAATTTC
T7-crRNA-gE46-R-GAC	cggtggagagGTCgtccATCTACAACAGTAGAAATTCCCTATAGTGA GTCGTATTAATTTC

Supplementary Table S3. Sequences of Cas12a proteins used in this study

Name	GI number	Species
FnCas12a	489130501	*Francisella tularensis*
AsCas12a	545612232	*Acidaminococcus sp*. BV3L6
LbCas12a	917059416	*Lachnospiraceae bacterium* ND2006
Lb5Cas12a	652820612	*Lachnospiraceae bacterium* NC2008
HkCas12a	491540987	*Helcococcus kunzii* ATCC 51366
OsCas12a	909652572	*Oribacterium sp.* NK2B42
TsCas12a	972924080	*Thiomicrospira sp.* XS5

BbCas12a	987324269	*Bacteroidales bacterium* KA00251
BoCas12a	496509559	*Bacteroidetes oral taxon* 274 str. F0058
Lb4Cas12a	769130406	*Lachnospiraceae bacterium* MC2017

Supplementary Table S4. SNP variants tested with HOLMES genotyping

ID	Gene	Category
rs5082	APOA2	Saturated fat consumption and weight gain
rs1467558	CD44	Acetaminophen metabolism
rs2952768	near CREB1	Morphine dependence
rs4363657	SLCO1B1	4.5x increase myopathy risk for statin users
rs601338	FUT2	Resistance to norovirus
rs6869366	TMEM167A	risk for bladder cancer
rs10034228		risk for pathological myopia
rs9939609	FTO	triggers obesity and type-2 diabetes
rs838133	FGF21	Higher odds of preferring candy
rs17070145	WWC1	greatly increased memory performance
rs6265	BDNF	brain-derived neurotrophic factor BDNF gene
rs1014290	SLC2A9	risk for gout
rs737267	SLC2A9	risk for gout
rs642803	OVOL1	risk for gout

Supplementary Figures

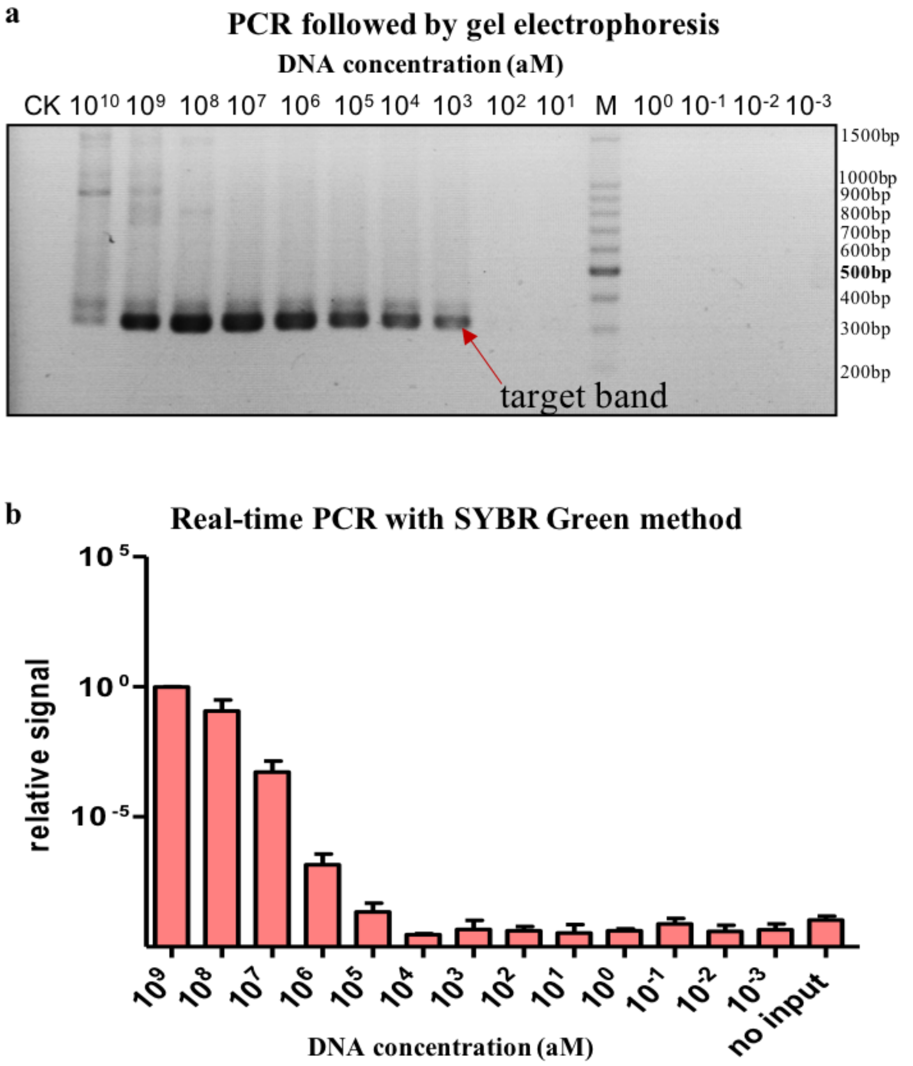

Supplementary Figure S1. Determination of the sensitivity of different nucleic acid detection methods. a, Determination of the sensitivity of PCR amplification. Serially diluted dsDNA (pUC18-T1) was used as the template, and the PCR products were analysed by gel electrophoresis and subsequent ethidium bromide staining. The same PCR-amplified samples were used to determine the HOLMES sensitivity (Figure 1c). The minimum detection concentration for PCR amplification by the KOD FX (ToYoBo) was 10^3 aM in this study. **b**, Determination of the sensitivity of quantitative PCR with the SYBR Green method. Serially diluted dsDNA (pUC18-T1) was used as the template. The minimum detection concentration for quantitative PCR with SYBR® Premix Ex Taq ™ II (TaKaRa) was 10^5 aM in this study. (n=3 technical replicates; bars represent the mean ± SEM)

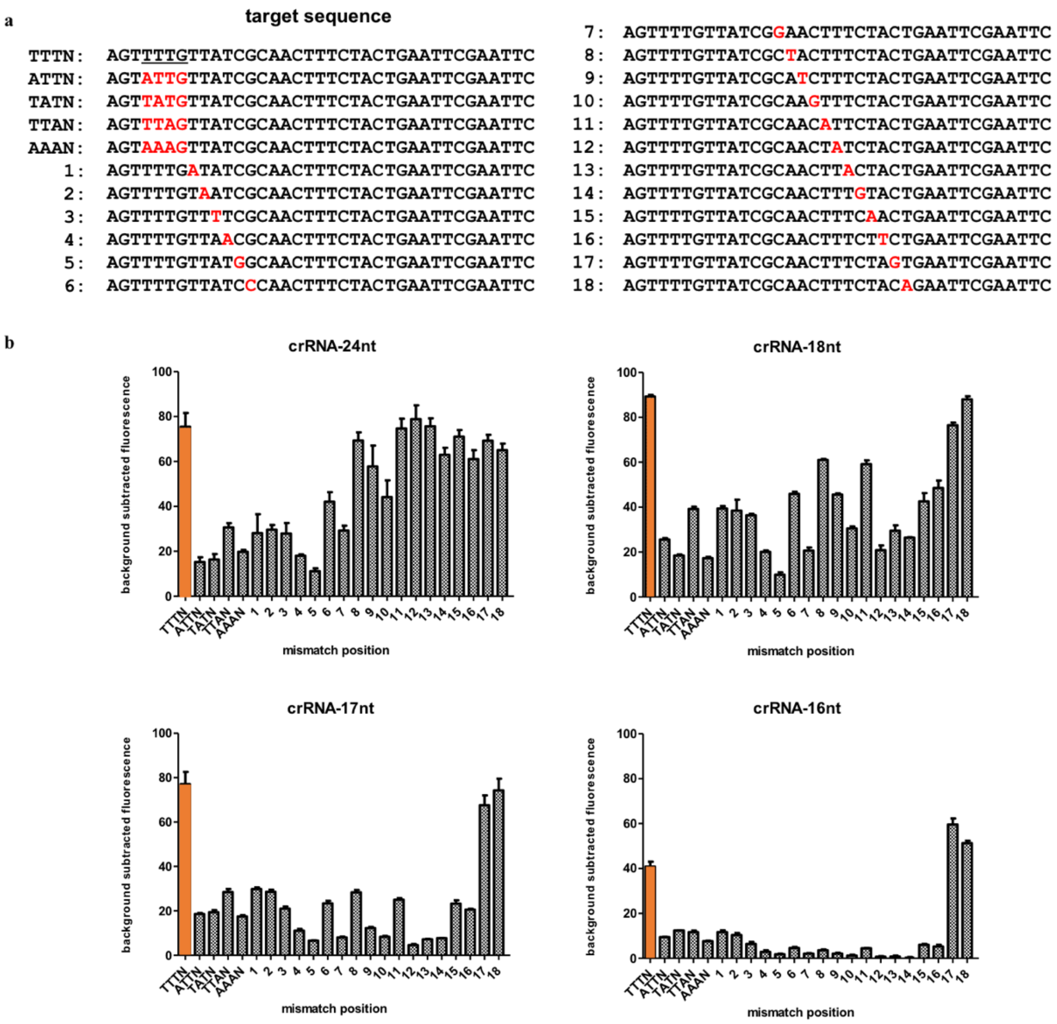

Supplementary Figure S2. Comparative analysis of the signal-to-noise of single-base mismatch with crRNAs of different guide lengths. a, Representation of target sequences in Supplementary Figure S2b. Target-T1 was employed for analysis. The PAM sequence in the wild type ("TTTN") was underlined, while mutated sequences were shown in red. **b**, Comparative analysis of the detection signal among target-T1 and its mutants shown above. crRNAs with different lengths of guide sequences were used. A quenched fluorescent DNA probe (HEX-N12-BHQ1) was incubated with the Cas12a reaction system, including LbCas12a, a non-mutated target-T1 ("TTTN"), a mutated PAM ("ATTN", "TATN", "TTAN" or "AAAN") or a sequence with a single base mutation from the 1st to the 18th base (No. 1-18) as the target sequence, and crRNAs with different lengths of guide sequences (*i.e.* crRNA-16nt, crRNA-17nt, crRNA-18nt, and crRNA-24nt) (n=3 technical replicates; bars represent the mean ± SEM). When the 24-nt crRNA complementary guide sequence (crRNA-24nt) was used, no difference was observed between the wild-type and single-base mutants altered in the region from the 8th -18th base positions; however, for the PAM mutants and mutants in region of the 1st -7th base positions, the fluorescence signal clearly

declined. When shorter guide lengths were used (*i.e.* 16-18 nt), the signals for wild-type and mutated targets differed more greatly. When 15-nt guide sequence was used, the HOLMES signals were very weak for target-T1 (data not shown), but were not for other targets, indicating the different characteristics among different crRNA guide sequences.

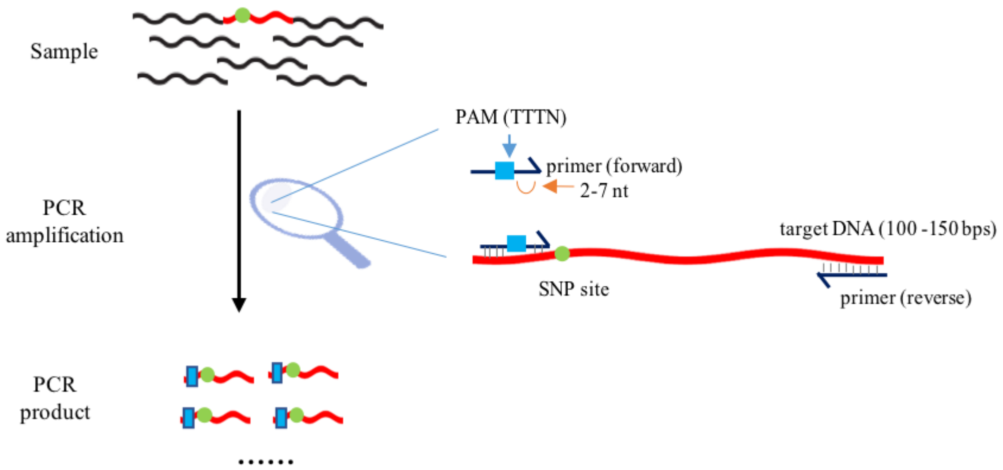

Supplementary Figure S3. Schematic of the primer design for SNP detection with HOLMES. Primers were usually designed to make sure that the SNP site located in the seed region (*i.e.* the first 8 nts) of the guide sequence. The PAM sequence ("TTTN") was contained in the primers and introduced into the target sequences through PCR amplification, enabling HOLMES to detect any SNP sites in a sequence-independent manner. Notably, the 3′-end pairing between the forward primer and the template was necessary for the successful amplification of the target region. Moreover, the length of the guide sequence in crRNA could be adjusted to achieve larger differences in fluorescence signals between different polymorphisms.

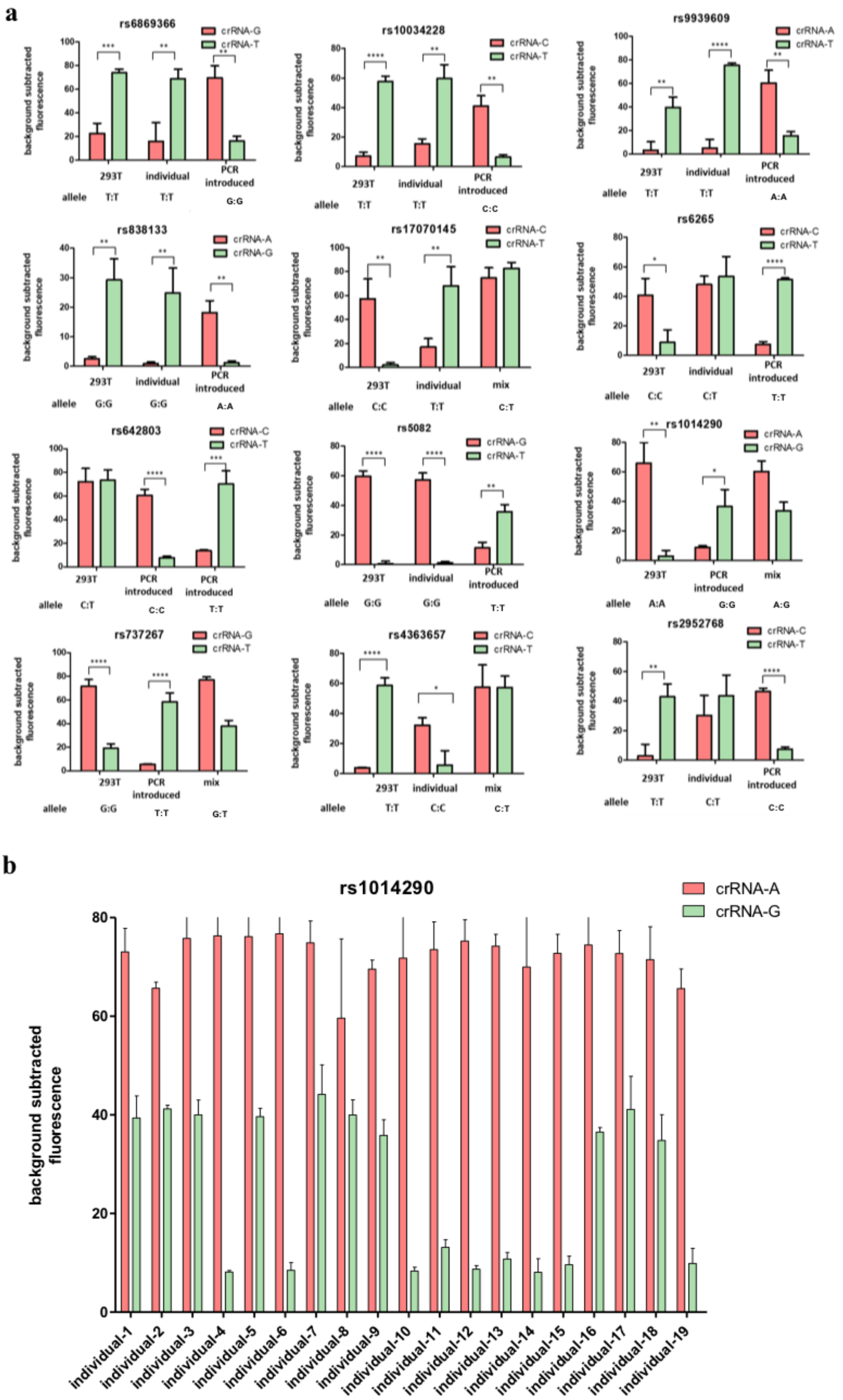

Supplementary Figure S4. Human SNP genotyping with HOLMES. a, HOLMES correctly genotyped 12 different SNP sites in the human genome. Genotypes verified by Sanger sequencing were

annotated below each plot. If the individual genotype was the same as that of HEK 293T, PCR amplification was employed to introduce a distinct genotype at the locus; otherwise, the PCR products of HEK 293T and the individual were mixed to mimic a heterozygous genotype. (n=3 technical replicates; two-tailed Student's t test; *, $p < 0.05$; **, $p < 0.01$; ***, $p < 0.001$; ****, $p < 0.0001$; bars represent the mean ± SEM) **b**, Detection of an SNP site (rs1014290) involved in the gout risk in 19 volunteers. A mixed SNP template mimicking a heterozygous genotype was employed as the control. As the signal value for crRNA-G with the mixed template of rs1014290 was obviously lower than that of crRNA-A (also see Figure S4a), signals for individuals were statistically analyzed with the control signal using the same type of crRNA (n=3 technical replicates; bars represent the mean ± SEM; two-tailed Student's t test; **, $p < 0.01$). Based on the HOLMES results, the genotype of volunteers 4, 6, 10-15 and 19 was A:A, while the rest was A:G, which was consistent with the Sanger sequencing results. This study was approved by the Biomedical Research Ethics Committee of SIBS, CAS, and only healthy human volunteers were used in this study.

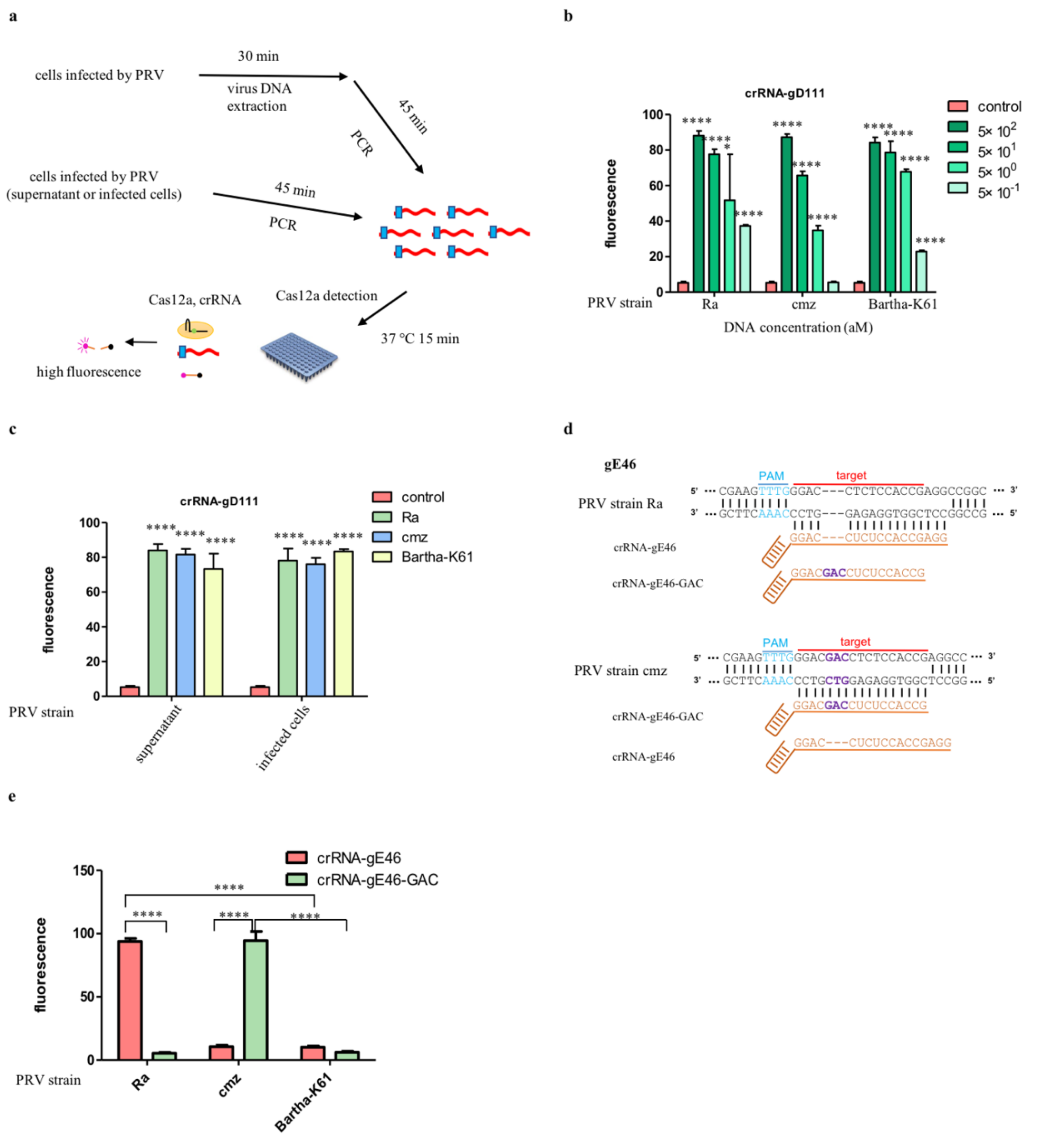

Supplementary Figure S5. Application of HOLMES in PRV virus detection. a, Schematic of PRV DNA virus detection by HOLMES. **b**, HOLMES detected PRV viruses with high sensitivity. **c**, HOLMES was of high sensitivity, and successfully detected PRV viruses in both the PRV-infected cells and the culture supernatant. **d**, Schematic of the target region in PRV strains and the crRNA sequences used for detection. Strain cmz had a small insertion of "GAC" in the region, which was shown in purple; whereas the Bartha-K61 vaccine strain lacked this target region. PAM sequences indicated in blue were introduced by PCR amplification with primers containing the PAM sequences, using the strategy illustrated in Supplementary Figure S3. **e**, Discrimination of the PRV Ra, CM and Bartha-K61 vaccine strains by HOLMES based on the strain SNPs. (n=3 technical replicates, two-tailed Student t test; ****, $p < 0.0001$; bars represent mean ± SEM)

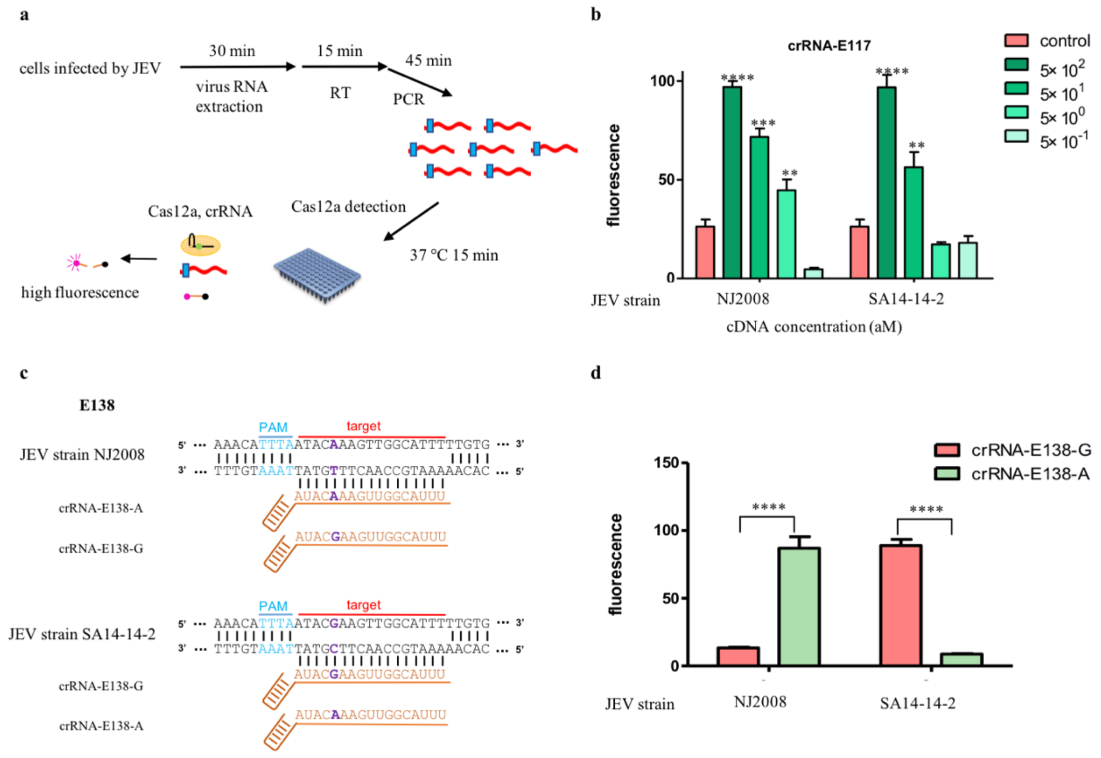

Supplementary Figure S6. Application of HOLMES in JEV virus detection. a, Schematic of JEV RNA virus detection by HOLMES. **b**, HOLMES detected JEV viruses with high sensitivity. **c**, Schematic of the targeted region in JEV strains and the crRNA sequences used for detection. SNPs in the target were coloured purple. PAM sequences introduced by PCR amplification were coloured in blue. **d**, Discrimination of JEV NJ2008 and SA14-14-2 strains by HOLMES based on the strain SNPs. (n=3 technical replicates; two-tailed Student's t test; ****, $p < 0.0001$; bars represent the mean ± SEM)

REFERENCES

1 Li, S. Y., Zhao, G. P. & Wang, J. C-Brick: A New Standard for Assembly of Biological Parts Using Cpf1. *ACS Synth Biol* **5**, 1383-1388 (2016).

2 Lei, C. *et al.* The CCTL (Cpf1-assisted Cutting and Taq DNA ligase-assisted Ligation) method for efficient editing of large DNA constructs in vitro. *Nucleic Acids Res*, **45**, e74 (2017).
